# Application of Dendrimer-based Nanoparticles in Glioma Imaging

**DOI:** 10.4172/2157-7439.1000444

**Published:** 2017-06-14

**Authors:** Sunalee Gonawala, Meser M Ali

**Affiliations:** 1 Department of Neurology, Henry Ford Hospital, Detroit, MI, USA; 2 Department of Chemical Engineering and Material Science, Wayne State University, Detroit, MI, USA

**Keywords:** Glioma, Tumor blood-brain barrier, Dual modality, CEST MRI, Optical imaging;

## Abstract

Dendritic polymers or dendrimers present an alternate template for the development of nanoparticulate-based drug delivery and imaging systems. The smaller size (~7–12 nm) of dendrimers have the advantage over the other particles, because its smaller size can possibly improve tumor penetration and the inclusion of tumor specific drug release mechanisms. A Paramagnetic Chemical Exchange Saturation Transfer (PARACEST) MRI contrast agent, Eu-DOTA-Gly4 or a clinical relevant Gd-DOTA was conjugated on the surface of a G5 PAMAM dendrimer. To create a dual mode MRI-optical imaging nanoparticle, Dylight680 was also incorporated on the amines surface of a G5 dendrimer. The particle was detected with *in vivo* MRI in preclinical glioma animal model. Furthermore, noninvasive imaging results were validated with in vivo and *ex-vivo* optical imaging.

## Malignant Glioma Imaging

Glioma is a central nervous system neoplasm that typically exhibits hypervascularity, especially grade III and IV tumors with marked heterogeneity. High rate of occurrence and intrusive phenotype leading to poor prognosis make malignant gliomas a major clinical problem. Gliomas represent 78% of all malignant brain tumors, and are the most prevalent cause for cancer-related death in males between age of 20–39 years [1]. Invasive biopsy is commonly used to assess the classification, histological type, grade and potential aggressiveness of brain cancer as well as to determine the type of drug regime utilized for treatment [2,3]. Noninvasive imaging techniques such as CT, ultrasound, PET and MRI are used for further confirmation [4–8]. However, for some brain tumors, the peritumoral edema does not easily provide for precise discrimination of tumor margins which makes the quantification of actual tumor volume difficult [9]. Therefore, the use of contrast agent may help overcome this deficiency and allow estimates of tumor margins from the largest cross-sectional area [10–12]. Specifically, we briefly discuss the applications of dendrimer-based nanoparticles for brain cancer imaging.

In clinical practice, T1 relaxation-based contrast agents are being used to discriminate tissue abnormalities in brain as well as other organs [13]. Specially, Magnevist (Gd-DTPA) has been used to generate positive contrast in T1-weighted images [14]. The major problem with low molecular weight agents such as Magnevist is that they extravasate from the blood into the interstitium of both normal and pathological tissues rather quickly, usually within 1–2 passes through the vascular bed. In glioblastoma (GBM), angiogenesis results in formation of torturous and irregular leaky vessels [15–17]. Therefore, this defective vasculature allows the nanoparticles to accumulate and be retained in tumor interstitium following systemic administration - a targeting strategy referred as Enhanced Permeability and Retention (EPR) effect [18]. Thus, if one had a nano-sized sensitive imaging agent that could be confined to the vascular bed of healthy tissues and become “leaky” only in rapidly proliferating tumors, then one should obtain specific contrast enhancement only in malignant tissue regions.

There are many ways a relaxation agent can be confined to the vascular bed including conjugation of a simple chelate such as DOTA or DTPA to high molecular weight macromolecules such as albumin, polylysine, or dendrimers of various sizes, prepare large nanoparticles such as iron oxide particles (SPIOs, USPIOs). Many of these larger particles present problems because they tend to be taken up via the liver or spleen. The pore size and the porosity of tumor vessels differ based on the tumor type and the status. Therefore, the effect of nanoparticle-based drug delivery differs based on tumor types, locations, sizes, grades and stages. Similarly, the in vivo effect of dendrimer-based Gd-DTPA contrast agents depends on the dendrimers core, size and the external surface charge [19,20]. Therefore, our lack of understanding about nanoparticle properties relative to the physiologic pore sizes within the blood-brain tumor barrier (BBTB), makes the distribution of nanoparticles across the BBTB a difficult task. Pharmacokinetic studies were performed across the BBTB in glioma tumor bearing rats, using different PAMAM dendrimer-based Gn-Gd-DTPA (n=1 to 8) nanoparticulate agents [18,21,22]. From the study, it was revealed that the core size of the gadolinium chelated dendrimers nanoparticles should be <12 nm to penetrate the BBTB, while the penetration of larger nanoparticles was deterred [23].

This suggests that the pore size in the BBTB of malignant brain tumors has a upper limit of approximately 12 nm [18,24,25]. Dendrimer-based spherical, paramagnetic nanoparticles with a diameter between 4 to 10 nm, maintain higher blood concentrations for considerable hours [18,23,26]. In previous studies, DTPA, a linear acyclic ligand, which is thermodynamically less stable, was used instead of macro cyclic ligand, DOTA to bind Gd^3+^ in the nanostructures. Secondly, although T1 weighted images that were collected using dendrimer-based Gd-DTPA, showed a statistically significant contrast enhancement in the glioma tumors, but these results were not confirmed using other imaging methods.

**Figure 1:**
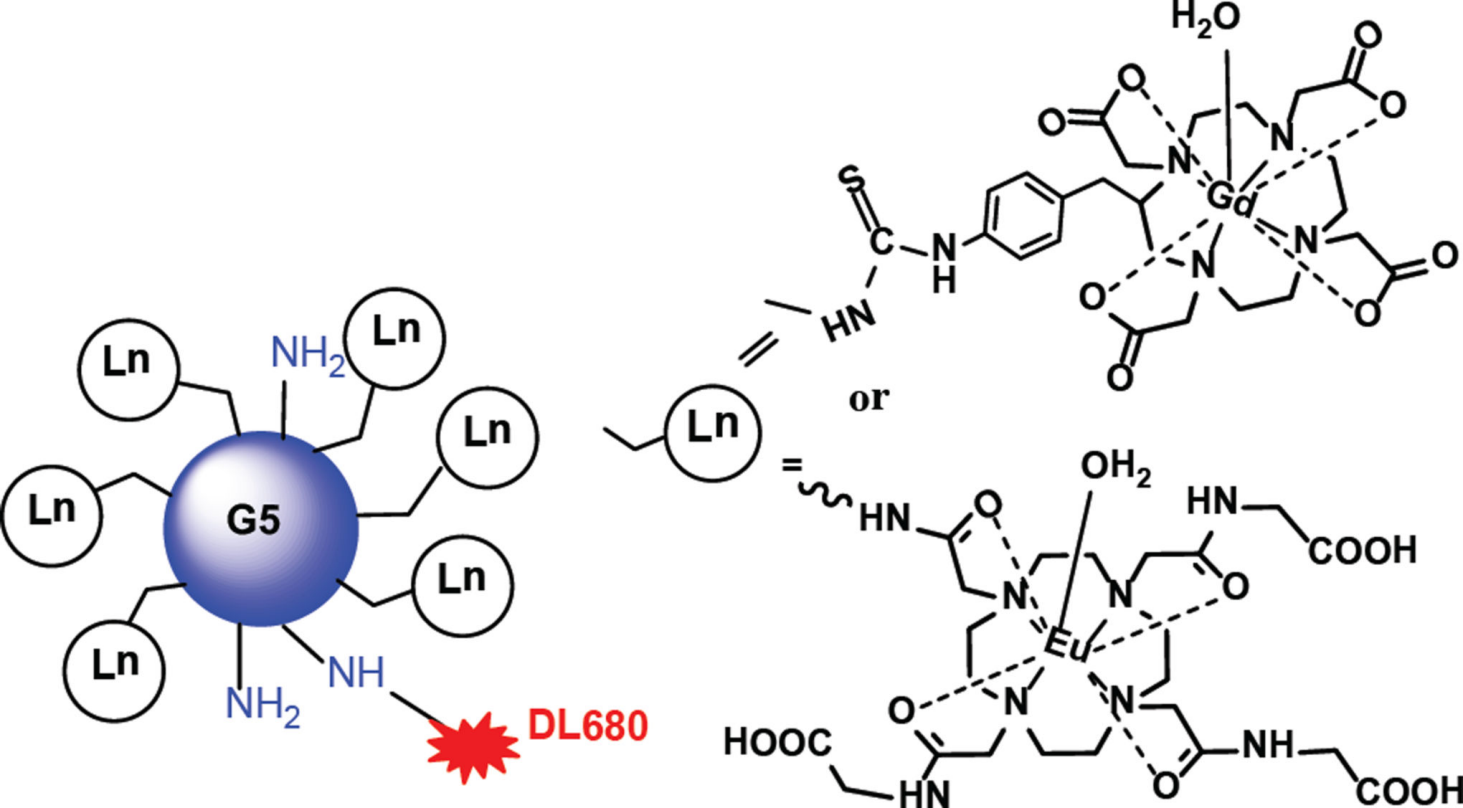
Schematic view of dual mode dendrimer-based agents. Functionalized Gd-DOTA or Eu-DOTA-Gly4 was conjugated on the surface of a G5 PAMAM dendrimer. DyLight 680 was also conjugated with Gd-DOTA or Eu-DOTA-Gly4 preloaded G5 dendrimer.

**Figure 2:**
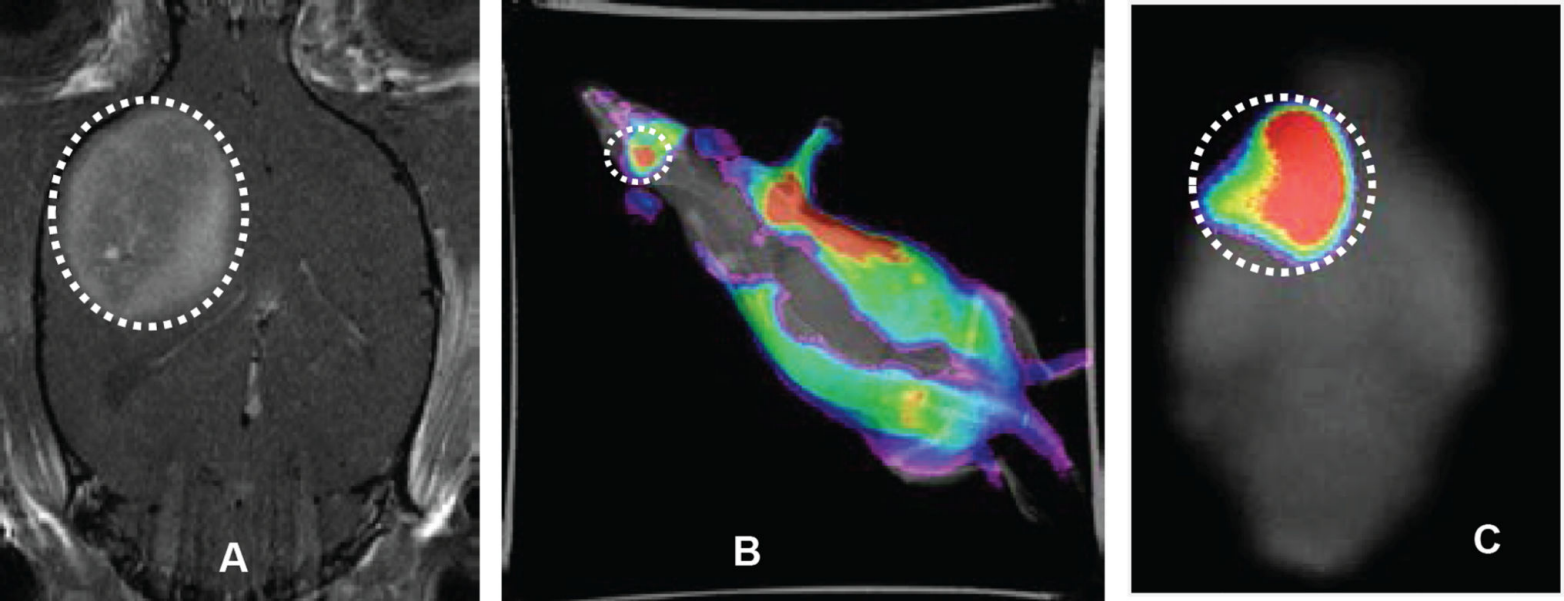
The coronal *in vivo* MRI image shows the location of U-251 glioma tumor (A). The agent was Gd-G5-DL680 and injected at a dose of 0.03 mmol Gd/kg. *In vivo* optical image obtained under simultaneous white light and filtered (540–690 nm) excitation detected with the emission filter set at 750 nm demonstrating fluorescence in the glioma (B). *Ex vivo* fluorescence imaging of rat brain clearly shows the selective accumulation of the Gd-G5-DL680 within the tumor (C). Tumor is indicated as dotted white circle.

**Figure 3:**
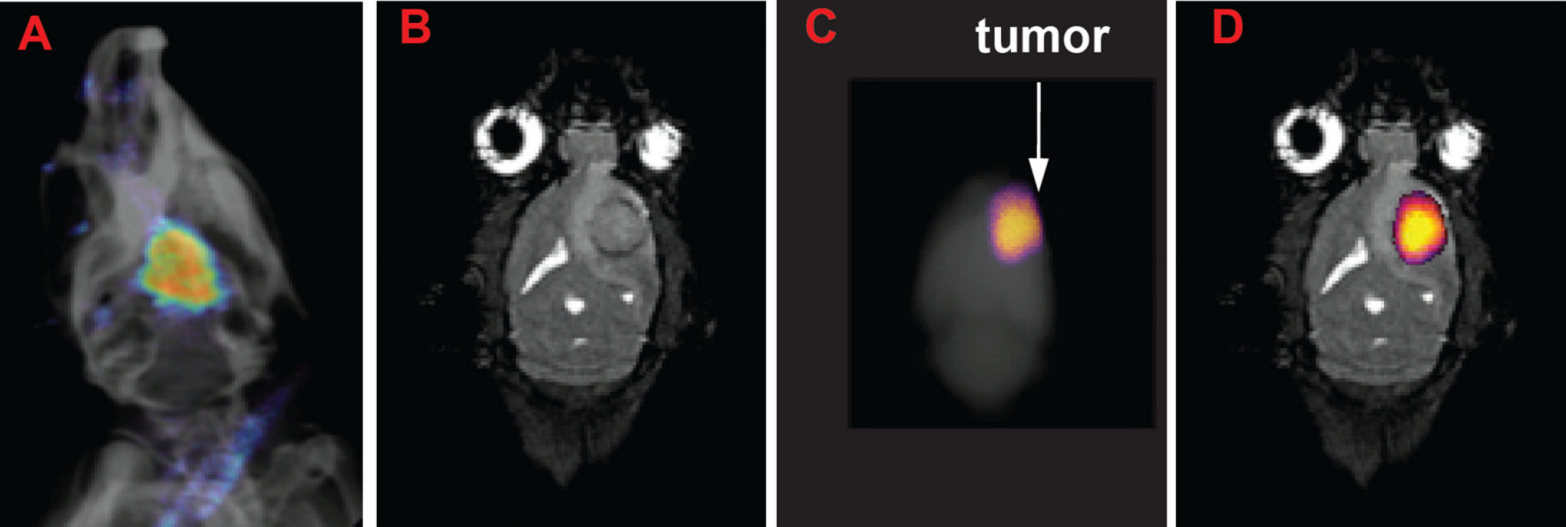
A) The *in vivo* fluorescent image of the rat head overlayed on an X-ray image shows the presence of Eu-DOTA-Gly4-G5-DL680 nanoparticle (NP) in the U87 tumor in the brain. B) The coronal MR image shows the location of the U87 tumor. C) The *ex vivo* fluorescence image of whole brain also detected the NP in the brain (fluorescent image was overlayed on X-ray image of the whole brain). D) The *ex vivo* fluorescence image was also overlayed on the MR image to show that the NP was located in the U87 glioma. The anatomy of the brain was correlated between MRI and X-Ray images.

Validation of imaging results can perform using dual-modality contrast agents, as the conditions that may affect the analysis of one imaging modality barely affect the other. Thus, these contrast agents can be assembled to take the advantage of two complimentary imaging modalities [17]. MRI is generally used to diagnose the macroscopic structures of pathological tissues in presurgical planning, whereas fluorescence imaging is used as a intrasurgical resource to diagnose microscopic information of pathological tissues [14,17,27]. Thus, a synergistic approach is to conjugate a fluorescent agent and a T1 relaxation agent or a PARAmagnetic Chemical Exchange Saturation Transfer (PARACEST) agent to the same dendrimer-based carrier. Taking this into account, a dual modality new contrast agent was synthesized by Karki et al. by incorporating clinically applicable MRI contrast agent, Gd-DOTA and a Dylight 680 with a generation 5 (G5) dendrimer (Figure 1) for glioma imaging (14). Similarly, a nano-sized, dual-modality imaging contrast agent was synthesized by conjugating a PARACEST MRI contrast agent, Eu-DOTA-Gly4, and a fluorescent agent, Dylight 680, to a G5-PAMAM dendrimer (Figure 1) [17]. Both *ex vivo* and *in vivo* imaging of the dual-modality dendrimer-based contrast agents showed exquisite possible utility for recognizing the location of gliomas, which demonstrate the benefit of using an exogenous contrast agent for glioma disclosure. Tumor location was identified at a millimeter scale due to the perseverance of the MRI contrast throughout the glioma (Figures 2 and 3) [14,17].

**Figure 4:**
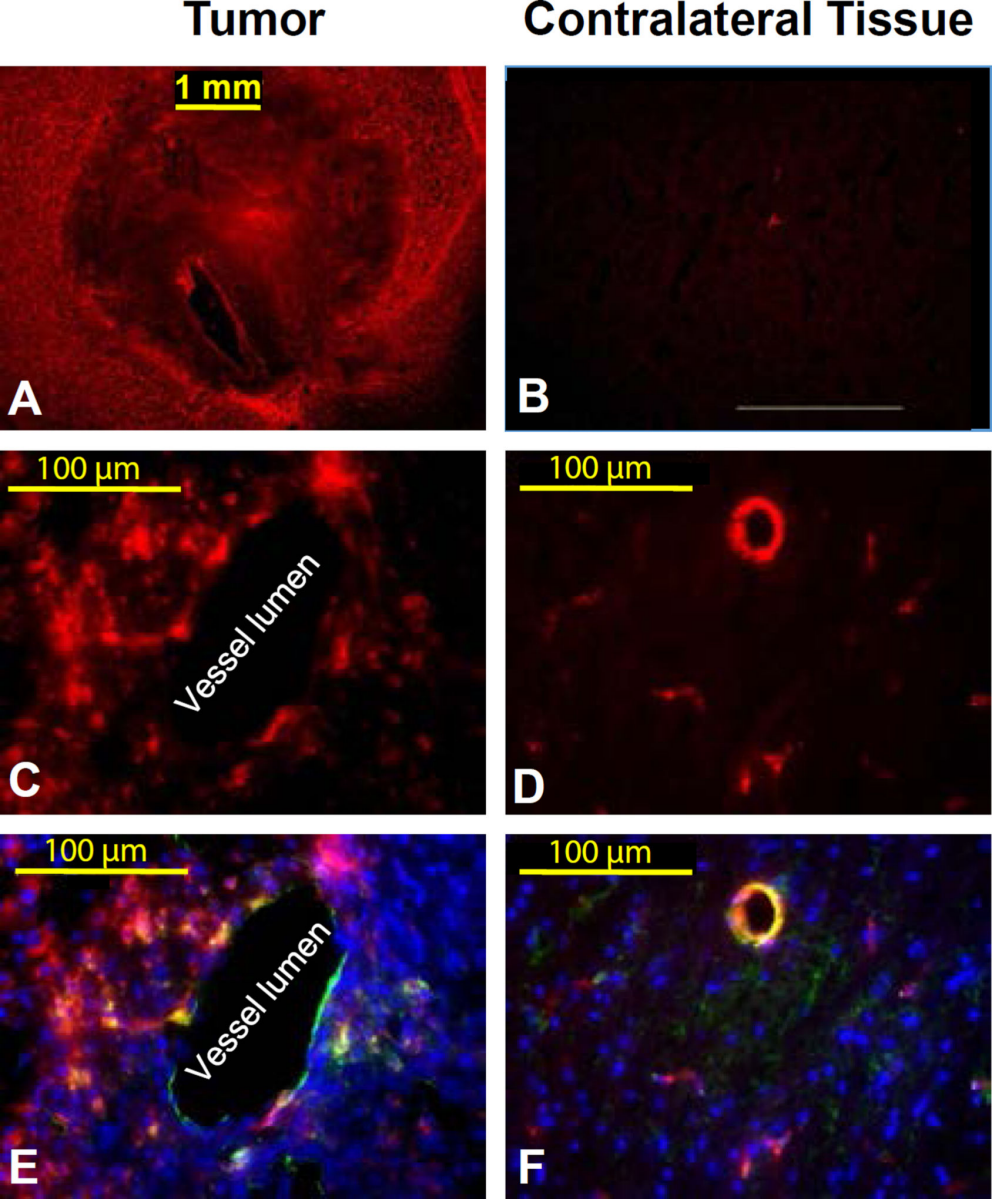
A) Strong, pervasive distribution of the agent was visualized in tumor tissue, B) and weak, focal distribution was visualized in the contralateral tissue. A high-resolution view showed that C) Eu-DOTA-Gly4-G5-DL680 nanoparticle extravasated across the endothlium in tumor tissue, D) but not in contralateral tissue. (E and F) Overlays of fluorescence from the agent, lectin staining of endothelium, and DAPI staining for viable cellularity validated the spatial distribution of the NPs observed in panels C and D. The exposure time for tumor area and contralateral brain was kept identical. The images of panels D and F were enhanced to show fluorescent activity in the vessels.

Furthermore, *ex vivo* fluorescence microscopy was used to validate the precision of *in vivo* MRI for glioma detection. Microscopy images displayed that the contrast agent concentrated in the glioma tumor, but not in the contralateral brain tissue (Figure 4A and 4B). Fluorescence microscopy at micrometer spatial resolution was performed ensuing lectin staining to identify vessel lumen and DAPI staining to identify viable cellularity (Figure 4C–4F). The multicolor microscopy images of the glioma indicated that the nanoparticles extravasated across the vessel lumen. Further, the images of the contralateral tissue demonstrated that no nanoparticles extravasate into normal brain tissues while they prevailed in the vessel lumen. This result further demonstrates that use of nanoparticulate dendrimer agents for the development of new imaging agents are more advantages in detecting gliomas [14,17].

Nonetheless, these successes with lipid-insoluble spherical nanoparticle formulations argues that a combination of targeted therapeutic approaches with nanoparticle formulations can be used to develop “active” dendrimer-based nanoparticles. Keeping this in mind, the surface of dendrimer-based carriers was modified with ligands that can specifically recognize the transferrin receptors that are overexpressed in endothelial cells in brain tumor tissue [28]. This strategy functions by interacting between the ligands (antibodies, peptide mimics, or nucleic acids) on the carrier surface and overexpressed receptors on the tumor cells [29]. However, these approaches have received very limited clinical success due to heterogeneous nature of cancer [30–32].

## Conclusion

In conclusion, a dual mode MRI-optical strategy is a useful method for in vivo biomedical imaging, as noninvasive high resolution anatomical images can be obtained using MRI, while microscopic details in post mortem pathological tissues can be obtained by fluorescence imaging.
